# Supplementary material for: The effect of intrapartum prolonged oxygen exposure on fetal metabolic status: secondary analysis from a randomized controlled trial
Source: Front Endocrinol (Lausanne). 2023 Jun 27;14:1204956. doi: 10.3389/fendo.2023.1204956 (PMC10335765; doi:10.3389/fendo.2023.1204956)
Supplement: Supplementary file 3 [file DataSheet_3.zip › Result-X101SC21092977-Z01-J001-B1-42/3.MetDiffScreening/O.vs.A/O.vs.A_neg_PCA.3D.pdf]

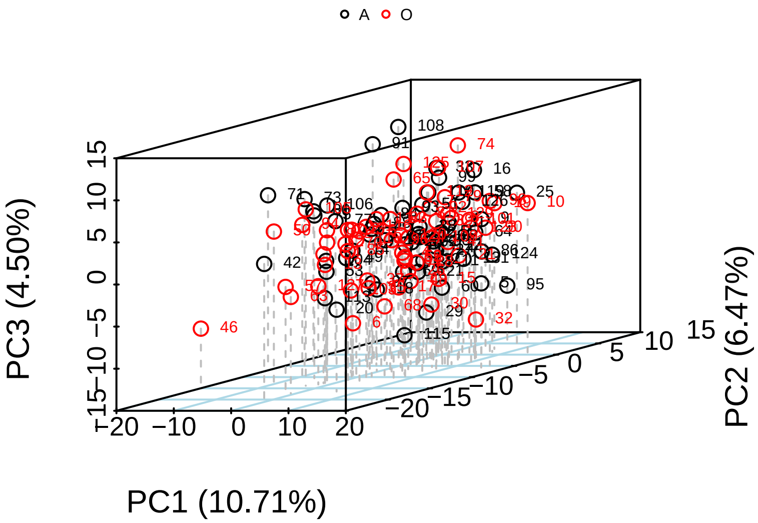

A
O
PC1 (10.71%)
PC2 (6.47%)
PC3 (4.50%)
−20 −10 0 10 20
−20 −15 −10 −5 0 5 10 15
−15 −10 −5 0 5 10 15
